# Ethical Aspects of Human Genome Research in Sports—A Narrative Review

**DOI:** 10.3390/genes15091216

**Published:** 2024-09-18

**Authors:** Aleksandra Bojarczuk

**Affiliations:** Biochemistry Department, Faculty of Physical Culture, Gdansk University of Physical Education and Sport, 80-336 Gdansk, Poland; aleksandra.bojarczuk@awf.gda.pl

**Keywords:** ethics, bioethics, genetic testing, sports, athletic performance, human rights

## Abstract

Human genome research in sports raises complex ethical considerations regarding the intersection of genetics and athletic performance. Pursuing genetic enhancements must uphold fairness, equality, and respect for human dignity. This narrative review explores the ethical dimensions of human genome research in sports, its potential implications on athletes, and the integrity of sports. As a narrative review, this study synthesizes the existing literature and expert insights to examine the ethical aspects of human genome research in sports. This study extensively examined the current literature on genetics, sports performance, ethical concerns, human rights, and legal regulations within the European context. The literature was searched using the SPORTDiscus, Scopus, Google Scholar, and PubMed databases. Exploring human genome research in sports reveals significant ethical implications, including potential genetic discrimination, impacts on human rights, and creating a genetic underclass of athletes. There are also definite benefits surrounding genetic testing. In conclusion, this review contends that integrating ethical considerations into developing and applying genetic technologies in sports is crucial to upholding fundamental principles of fairness, equality, and respect for human dignity. It stresses the importance of open and inclusive dialogue about the potential consequences of genetic advancements on athletic performance, future generations, and the integrity of sports.

## 1. Introduction

The pursuit of identifying athletic talent and the ethical challenges in sports genetics is a topic of increasing importance and controversy within the scientific community. Understanding the genetic underpinnings of athletic performance has led to significant advancements in molecular biology and genetics, offering new avenues for talent identification and performance enhancement in sports. This has raised complex ethical considerations regarding fairness, equality, and the well-being of athletes. The intersection of genetics and sports introduces profound ethical dilemmas that require careful consideration and ethical guidance to ensure the integrity of sports and the preservation of equal opportunities for all athletes. The current state of research in sports genetics has advanced significantly, with critical publications highlighting the influence of genetics on athletic performance, talent identification, and the ethical implications of genetic enhancements in sports. Controversial hypotheses have emerged regarding the ethical implications of genetic modifications, including concerns about genetic discrimination, the creation of a genetic underclass of athletes, and the potential impact on future generations. Concurrently, genetic testing can elevate athletic performance and significantly advance sports and human capabilities. These diverging viewpoints underscore the need for a comprehensive ethical framework to guide the responsible development and application of genetic technologies in sports.

## 2. Methods

The literature search was conducted across four databases, SPORTDiscus, Scopus, Google Scholar, and PubMed, from 26 June 2024 to 14 August 2024, using Boolean operators AND/OR to refine the results. The search was guided by keywords such as “genetics“, “sports performance“, “ethics“, “bioethics”, “genetic testing”, “genetic doping”, “human rights”, “sports”, and “legal regulations”. Inclusion criteria were set to ensure the relevance and comprehensiveness of the gathered works, including full-length research articles, review articles, book chapters, and shorter pieces like letters, editorials, and commentaries that offered focused insights or opinions. To be included, articles had to be directly related to the specified topics and available as full text, with English language content being a requirement. The search strategy was designed without specific time constraints, allowing for incorporating both recent and seminal works in the field. Exclusion criteria were applied to eliminate non-human studies, such as those focusing solely on animal research and conference abstracts or papers not available as full text.

## 3. Results

### 3.1. Evolution and Athletic Talent and Identification and Ethical Challenges in Sports Genetics

The pursuit of identifying individuals with natural talent for sports is influenced by evolution, which selects favorable and unfavorable traits for specific sports. Successful talent selection involves accurately predicting the evolving trends in particular movements. This helps anticipate how the skills and competencies essential for successful performance will change between the selection process and the demonstration of elite skills [1,2]. However, it is essential to recognize that talent selection involves more than evolutionary traits. It also includes factors like training, environment, and individual effort. Moreover, many highlight that talent has genetic roots. It is widely accepted that genetics play a significant role in human athletic ability. So far, 251 polymorphisms have been pinpointed as associated with athletic performance through candidate genes and GWAS studies, showcasing technological progress and its potential [3]. The literature claims that the coming years will be an exhilarating time for sports genomics, marked by advancements in DNA technologies such as whole-genome sequencing, genome-wide association studies (GWASs), epigenomic studies, transcriptomic analysis, proteomic profiling, and bioinformatics. This progress will delve deeper into and evaluate the genetic impact on human physical performance. Human genome research will be enhanced by the growing access to comprehensive omics databases and high-throughput screening methods for studying human performance. The future holds great promise by incorporating multi-omics approaches that combine data from different omics levels (e.g., genomics, metagenomics, epigenomics, transcriptomics) to comprehend their interconnection and collective influence on the biological mechanisms of physical performance [4,5]. While ethical considerations surrounding genetic technologies in sports are essential, it is equally vital to recognize their substantial benefits, particularly regarding talent identification and performance enhancement. Much literature has been published on this [3,5,6,7,8]. For instance, personalized training programs based on genetic information can lead to more effective and tailored workouts, enhancing strengths and addressing potential weaknesses to improve overall performance [9]. Genetic technologies can also enhance injury prevention and recovery. These technologies analyze genetic markers related to tissue repair, injury susceptibility, and recovery times to develop personalized strategies for minimizing the risk of injuries and enhancing recovery processes. This ensures a more practical approach to maintaining athlete health and extending careers [10]. Next, understanding an athlete’s genetic predisposition for metabolism and nutrient processing allows for developing tailored dietary strategies that enhance energy levels, optimize muscle growth, and improve overall health. Personalized nutrition plans can help athletes achieve peak performance by ensuring that they receive the nutrients best suited to their genetic profiles [11]. Lastly, genetic technologies have the potential to democratize talent identification by providing access to insights previously available only to elite athletes. As genetic testing becomes more accessible and affordable, more athletes can benefit from personalized feedback and development opportunities. This can lead to a more inclusive sports environment where talent is recognized and nurtured regardless of socio-economic background [12]. However, the current value of DNA testing for identifying talent or sports performance phenotypes is likely to be limited [13]. Despite the obvious role of genetics in human athletic performance, there is limited conclusive evidence supporting a specific genetic variation that significantly affects athletic performance within the normal range of human traits. This could be due to the complexity of traits being influenced by multiple genes with minor effects and researchers’ oversight of environmental factors. It is important to understand that individual DNA characteristics likely only account for a minimal percentage of overall physical traits, typically ranging from 0.005% to 0.1% [14]. Furthermore, the literature argues that genomic testing is not a valuable tool for identifying talent. One study compared the combined genotype scores for the endurance (68 genetic variants) and speed–power (48 genetic variants) elite athlete status of five elite track and field athletes, which included an Olympic champion, with 503 non-athletic Caucasian controls. The results indicated that elite speed–power athletes had higher scores using the speed–power total genotype score than elite endurance athletes. However, 68 non-athletic controls had even higher scores than elite power athletes using this score. Surprisingly, elite speed–power athletes also had higher endurance–total genotype scores than elite endurance athletes. The study concluded that genetic information is unreliable in distinguishing between elite athletes and non-athletic controls, thus questioning its effectiveness as a talent identification [15]. Interestingly, the perspectives of athletes and coaches on genetic testing are various. The prevalence and attitudes towards genetic testing were examined in a study involving 243 participants, including 110 athletes and 133 support staff from across the globe. The study found that only 10% of athletes and 11% of support staff had undergone genetic testing. Most of the participants believed that genetics played a role in athletic success and training adaptation. The main barriers to genetic testing were the need for more awareness, high cost, and insufficient scientific evidence. However, 73% of athletes and 64% of support staff who underwent genetic testing found their information helpful. Furthermore, most individuals who have used genetic testing in sports use it not to identify talent but to help design training programs. Only 4% of athletes mentioned ethical considerations for not utilizing genetic testing in the survey forms, compared to 19% of coaches [16]. This indicates a range of attitudes towards its application and usefulness. Ethical concerns are more prominent among coaches than athletes, suggesting differing views on the implications of genetic testing within the sports community. In another study, 72 elite athletes and 95 support staff from UK sports clubs and governing bodies were surveyed online about their experiences with genetic testing and their attitudes towards its use in sports. Most elite athletes (about 80%) and nearly all their support staff believed that genetics was crucial in determining athletic abilities. They also believed genetic profiling could be valuable for predicting athletic performance and injury susceptibility to injuries [17]. Next, the validity and predictive power of genetic tests for talent are criticized based on the ACCE model, which reviews analytic and clinical validity, clinical utility, and ethical, legal, and social implications. While competent and ethically sensitive talent identification can be justified, the competence requirement does not encompass genetic tests [18]. In addition, genetic testing in individuals under 18 raises ethical concerns, with some researchers advising against it [19,20,21]. Genetic testing in minors presents unique challenges and ethical dilemmas beyond the scope of general competence and validity assessments, highlighting the need for careful consideration before implementing such tests in young athletes.

However, one of the significant ethical issues related to genetics in sports involves performance-enhancement methods that do not directly modify genetic material [22]. For example, genetic testing can be used to select athletic talent. Some interventions involve direct human genome modification, such as somatic gene transfer techniques, and more extreme methods, like sex line modification. Germline modifications can create athletes with inherent physical advantages that last throughout their lives, offering a significant edge in competitive sports. In addition, beneficial traits can be passed on to future generations, potentially leading to a lineage of genetically enhanced athletes with superior physical capabilities [23]. Somatic gene therapy involves inserting healthy genes into non-reproductive cells to treat genetic diseases. The introduced genes do not affect future generations, meaning any changes produced by the therapy will be limited to the patient and not passed on to their children. The therapeutic genes have a limited lifespan as they are only present in the targeted cells, continuously replacing new cells [24]. Somatic genetic modification can, therefore, attenuate a medical condition and improve the athlete’s performance [25,26]. However, the implications of these advancements extend far beyond the individual athlete, raising profound concerns regarding human rights and the ethics of genetic enhancement in sports. The potential for genetic discrimination and the creation of a genetic underclass of athletes who lack access to such enhancements highlight the need for robust legal regulations and international standards. It also compromises the fairness of the gaming spirit of athletes. Such developments challenge the fundamental equity and fairness principles underpinning competitive sports. There is a risk of exacerbating inequalities between athletes who can afford genetic enhancements and those who cannot, diminishing the level playing field essential for genuine competition. To address these concerns, establishing comprehensive guidelines could ensure that all athletes have equal opportunities and preserve the spirit of fair play. Such measures could address the technical and ethical dimensions and promote transparency and inclusivity within the sporting community. Moreover, the permanence of germline modifications and their impact on future generations necessitate carefully considering our moral responsibilities towards those who will inherit these changes. These concerns underscore the importance of integrating ethical considerations into the development and application of genetic technologies in sports, ensuring that the pursuit of enhanced performance does not compromise the fundamental principles of fairness, equality, and respect for human dignity. As the scientific community continues to explore the potential of genetic modifications, engaging in an open and inclusive dialogue about the long-term implications of these technologies for the integrity of sports and society is crucial. The ethical aspects of human genome research in sports are paramount in considering the potential consequences of genetic advancements on athletic performance [27]. The intersection of genetics and sports introduces complex ethical considerations surrounding fairness, equality, and the well-being of athletes. It is essential to explore the ethical dimensions of human genome research in sports to ensure that the pursuit of genetic enhancements upholds the principles of fairness, respect for human dignity, and the preservation of equal opportunities for all athletes.

Additionally, the long-term implications of genetic modifications in sports warrant a comprehensive ethical framework to guide the responsible development and application of genetic technologies. Ethical inquiries into human genome research in sports will play a vital role in addressing the potential impact on athletes, future generations, and the integrity of sports. Therefore, this review explores human rights, legal regulations, and ethical considerations regarding genetic research’s current and potential implications in sports.

### 3.2. Human Rights and Legal Frameworks

Given biomedicine’s potential challenges, the dialogue regarding protecting human rights in Europe is of considerable significance across the European nations. The progress in genetic understanding has provided new avenues for identifying and analyzing specific genes and entire genomes and altering them [28,29]. This has implications for individuals and society. The development of genetics has triggered discussions, both domestically and internationally, about the ethical quandaries and the quest for moral grounds to support such research [30]. Bioethics is the scholarly discipline that addresses these ethical and moral concerns [31,32]. The findings stemming from genetic research necessitate adjustments in legal frameworks to keep pace with the evolving knowledge about the functions of individual genes [33].

It is essential to note that human rights protection primarily relies on the domestic legislation of individual countries [34,35,36,37]. While international treaties and agreements set standards, the actual implementation and protection of human rights depend on each nation’s legal framework and political will. As a result, the effectiveness of human rights protection differs widely from one jurisdiction to another, depending on each country’s dedication to these norms. In Poland, the Constitution of the Republic of Poland (Article 39), the Act on the Profession of Physician and Dentist (Article 25), the Regulations of the Minister of Health regarding Good Clinical Practice, and the Penal Code (Articles 22, 24, 27, 39) as well as the Code of Medical Ethics are the fundamental legal acts regulating research on humans. Furthermore, the European system for the protection of human rights, which includes the European Convention on Human Rights (ECHR) and the European Court of Human Rights in Strasbourg, plays a crucial role in overseeing the adherence to legal acts at the supranational level. This system operates under the Council of Europe and the European Union, which collaborate to create and enforce the European human rights protection system [38]. Within the Council of Europe, biomedicine is addressed by the Parliamentary Assembly, the Committee of Ministers, and the Steering Committee on Bioethics [39,40].

Similarly, the European Union deals with biomedicine through the European Parliament and the European Group on Ethics in Science and New Technologies, which also addresses the development of new information technologies [41]. Previously, Working Groups handled ethical and legal issues related to biomedicine, focusing on various aspects, such as research on human genome analyses [42]. From this arose the advisory group on ethical implications of biotechnology, known as the European Group on Ethics in Science and New Technologies, which functions as a permanent advisory body of the European Union.

The Convention on Human Rights and Biomedicine [43,44] involves a critical document regarding human rights protection in biomedicine. This convention and its additional protocols provide a comprehensive framework for the ethical application of biology and medicine in Europe. It addresses a wide range of issues, including the prohibition of human cloning, organ and tissue transplantation, and biomedical research [45]. The Biomedicine Convention was established because of the Council of Europe’s efforts in biomedicine, covering legal aspects related to transplantation, medical data protection, patients’ rights, biomedical research involving fetuses and embryos, genetic research, and human genomes. These areas encompass genetic screening, pre-implantation, prenatal testing, DNA material use in criminal proceedings, and patenting human genes and related inventions [46].

The regulations of the Biomedicine Convention establish guidelines for carrying out scientific research in the fields of biology and medicine. Also known as the Convention on Human Rights and Biomedicine or the European Bioethics Convention (EBC), the Convention for the Protection of Human Rights and Dignity of the Human Being concerning the Application of Biology and Medicine was adopted on 4 April 1997, in Oviedo. This convention aims to address the potential risks associated with uncontrolled advancements in biotechnology. It seeks to safeguard individuals from unauthorized interventions on their bodies, prevent the commercialization of the human body, and regulate the misuse of genetic testing, among other objectives. By protecting the genetic identity of every human being, the EBC also safeguards future generations’ well-being and humanity’s welfare. The EBC permits genetic intervention only for preventive, diagnostic, or therapeutic purposes and explicitly prohibits modification to the offspring’s genome [47]. The rapid progress in biomedicine offers promising solutions for human health issues and raises ethical and moral concerns. The challenge lies in applying newly acquired knowledge responsibly and determining the ethical boundaries of scientific research involving humans [35,48,49].

Recent advancements in human biology and medicine have prompted global discussions regarding imposing restrictions on human research. While therapeutic and scientific experiments share a common objective of advancing scientific knowledge, distinctions have been acknowledged. A therapeutic experiment aims to enhance health outcomes [50]. In contrast, scientific experiments are typically carried out to address specific research questions or test hypotheses. Their primary goal may not be to benefit the participants’ health directly. These experiments can involve studying healthy individuals to understand fundamental biological processes or the impacts of different interventions. The focus is often on collecting data to advance scientific knowledge rather than offering immediate therapeutic advantages [51]. The use of scientific knowledge in the field of genetics, particularly human reproductive cloning, has been a highly contentious issue and was officially prohibited on 12 January 1998. This ban was formalized through the Additional Protocol to the Convention on Human Rights and Biomedicine. The protocol explicitly forbids any intervention intended to create a human that is genetically identical to another, marking a significant step in international bioethics and the regulation of genetic technologies [52].

### 3.3. The Role of International Declarations: The Human Genome Project and Its Ethical Implications

Molecular genetics is a rapidly advancing field in medical science. From 1990 to 2003, the Human Genome Project [53], involving 26 countries, focused on studying the genetic information within the human genome. While the Human Genome Project did not directly influence sports, its contributions to genetic research have led to positive and controversial applications [54]. The Project provided fundamental information about the human blueprint. However, genetics have unlocked the potential for gene manipulation to enhance or diminish specific functions within the human body, which could be utilized to improve athletic performance. For example, altering genes to promote erythropoietin production to increase muscle mass and improve sports performance is a prospect that some find appealing. Still, the Project emphasizes the need for ethical oversight and the importance of protecting genetic information from misuse, particularly in light of rapid advancements in genetic engineering. This international effort was made possible by adopting the Universal Declaration on Human Rights and the Human Genome in 1997, acknowledging the genome as the “common heritage of mankind”. This concept was formally emphasized in the Universal Declaration on the Human Genome and Human Rights, adopted by The United Nations Educational, Scientific and Cultural Organization (UNESCO) in 1997, which recognized the human genome as part of the “heritage of humanity” [55,56].

The human genome is a collection of human genes and is often considered a specific form of knowledge [57]. The human genome refers to the complete set of genetic information encoded in the DNA of our species. It contains all the necessary information to build and maintain a human being. The human genome comprises over 3 billion DNA base pairs and contains genes that encode proteins and non-coding regions with regulatory functions [58]. The sequencing of the human genome has provided a wealth of knowledge about the genetic basis of human biology, which has dramatically advanced our understanding of health, development, and disease [59]. The Universal Declaration of Human Rights and The United Nations Convention, established on 5 June 1992, safeguard the diversity of humans and their genomes, which are subject to natural evolution [60]. At the very beginning of the project, to learn about the human genome, an advisory body, Ethical, Legal, and Social Implications (ELSI), was established in parallel to consider the problem of possible consequences of human genome research, intellectual property of the obtained genetic information, and the potential use of this information for other scientific disciplines [61,62]. The rapid advancements in genetic engineering and artificial intelligence research intertwine with the issue of safeguarding the genome. The potential to alter genetic makeup using the tools available through genetic information manipulation could have far-reaching implications for future generations [63]. These alterations may result in unpredictable consequences for individuals and humanity [64,65].

### 3.4. Ethical Considerations on Genetic Doping in Sports

The potential to alter genetic makeup using advanced technologies poses ethical challenges. The rapid progress in genetics and artificial intelligence could lead to far-reaching consequences, necessitating a careful approach to applying genetic knowledge. Ongoing research in human genome manipulation encompasses gene therapy [66], including the non-therapeutic application of genes and genetic elements to enhance athletes’ physical abilities, known as genetic doping [67,68,69]. The World Anti-Doping Agency (WADA) prohibited this practice in 2003 [70,71,72,73]. Established by the International Olympic Committee in Canada, WADA is dedicated to advancing, coordinating, and overseeing efforts to combat drug use in sports. WADA is entrusted with the World Anti-Doping Code, which has been embraced by over 650 sports organizations, including international sports federations, national anti-doping organizations, the International Olympic Committee, and the International Paralympic Committee. The World Anti-Doping Code (Code) is the fundamental document that standardizes anti-doping policies, rules, and regulations within sports organizations and public authorities worldwide. It operates with eight international standards designed to promote uniformity among anti-doping organizations in various areas.

Currently, two main methods of genetic doping are identified: (1) stimulating gene expression to increase the number of cells in the body, particularly those responsible for muscle development, and (2) implanting foreign tissues that multiply in the body [74,75,76].

In addition, the literature suggests that it is also possible to provide the athlete with specific modified bacteria that affect the production of hormones (e.g., growth). For example, bacteria can be engineered to produce growth hormone (GH), which is critical for muscle growth and recovery [77]. Therefore, administering modified bacteria represents a novel and advanced method of performance enhancement. This approach leverages genetic engineering to manipulate the body’s natural processes in a way that traditional doping methods cannot.

Gene doping in sports involves the use of factors such as human growth hormone (HGH) and insulin-like growth factor 1 (IGF-I) to increase strength and muscle mass [78], erythropoietin (EPO) to boost red blood cell count [74], myostatin inhibitors to enhance skeletal muscle mass [79], vascular endothelial growth factor (VEGF) to influence angiogenesis, and endorphins to produce an analgesic effect [80].

Doping opponents argue that it carries health risks, reduces the opponent’s chances and freedom, undermines the sporting spirit, creates negative role models, and desecrates human nature. In contrast, proponents of genetic doping might present several ethical arguments in its favor [81,82]. It cannot be ignored that doping could enhance an athlete’s autonomy by providing greater control over physical capabilities, allowing individuals to push the boundaries of their performance. Additionally, genetic doping could level the playing field by allowing athletes who face natural disadvantages to compete more equitably with their peers. This perspective also aligns genetic enhancements in sports with similar interventions in other areas of society, such as medicine and technology, where advancements are often embraced for their potential benefits. Furthermore, the remarkable achievements enabled by such enhancements might foster admiration and a sense of heroism, inspiring awe and respect for athletes who push the limits of human potential. Thus, while there are significant concerns about the ethical implications of genetic doping, these arguments highlight the complexity of the debate.

### 3.5. Ethical Debate on Performance Enhancement

The main objective of sports competitions is to measure, compare, and rank participants based on their athletic performance. A wide range of genetic and environmental factors, starting from the moment of conception to the actual performance, influence athletic performance. Some of these factors are random, such as timing and opportunities. Other factors, such as growing up in a sports-friendly or non-sporting environment or the chance of an unexpected wind carrying a javelin further during competition, involve elements of luck. Lastly, certain aspects of athletic performance are based on the athletes’ hard work and efforts to develop their talents [83].

The debate surrounding using gene transfer techniques to enhance sports performance has supporters and critics. Opponents argue that sports should be a domain for nurturing natural talent to achieve human excellence in line with social and moral ideals, expressing skepticism about biotechnological enhancements in sports. On the other hand, proponents question why we should doubt performance-enhancing technologies if they can contribute to human flourishing in and out of sports. This challenges our biases and attitudes. While technology in sports may not solely be about objective progress and breaking records, opponents’ perspective still holds sway. Whether these ideas can endure in a world where biotechnological enhancements in sports are on the rise remains to be seen. However, it should be remembered that sport, in its simplest form, is one of the most extraordinary human activities. It imposes rules that provide equal opportunities for all, such as entertainment and enjoyment, and allows participants to extend their physical and mental limits and share common values and experiences. Thus, in the discussion on whether doping should be considered unethical in sports, the reasons why certain substances or methods enhancing physical performance are banned should be considered [84]. Substances or methods significantly strengthening an athlete’s performance are strictly prohibited. Such enhancements distort athletes’ natural abilities and can lead to unfair advantages. Organizations like the WADA are dedicated to upholding the integrity of sports by ensuring that competition results reflect genuine athletic prowess, free from any artificial enhancements.

Additionally, banned substances and methods also include those that pose significant health risks to athletes, as the well-being of athletes is of utmost importance. Furthermore, any substances or methods that undermine the ethical foundation of sports are strictly prohibited, as they go against the values of fair play and respect that are fundamental to sportsmanship. Legally, the Declaration and convention have agreed to safeguard the human genome’s natural state. Our limited knowledge of the human genome influences this caution. There are opposing opinions on this issue, with some arguing against scientific advancements in genetics. The European Bioethics Convention permits only interventions that do not impact heredity and are focused on prevention, diagnosis, or therapy [85,86]. The Human Genome Project has allowed us to decipher the data encoded in human genes [87]. Ongoing research explores DNA analyses concerning potential health predispositions and genetic background (such as ethnicity and family history), raising valid concerns about discrimination [88]. Furthermore, it could enable the selection of individuals to enhance favorable hereditary traits and eliminate unfavorable ones.

This type of segregation in sports involves the potential selection of individuals based on their genetic profile suited for a specific physical activity such as speed or endurance. However, solely focusing on genetic factors and creating a “champion model” for a sport neglects the impact of environmental factors, willpower, and ambition and does not ensure championship success [89,90]. In addition, environmental factors such as access to training facilities, coaching, and support systems play a crucial role in an athlete’s success. Dedication and ambition are also significant determinants of championship success as they drive athletes to push their limits, overcome obstacles, and continually improve their performance. Therefore, a holistic approach that considers genetic predispositions, environmental influences, and the athlete’s mindset and determination is essential for predicting and cultivating championship success in sports [91,92]. Recent studies have also highlighted the limitations of genetic testing, as the association of specific genetic variants with certain physical traits does not fully determine athletic success [21,93]. Meanwhile, research suggests that approximately 10% of athletes have undergone genetic testing, and more would be willing to do so [94]. Determining an individual’s predisposition for a specific physical activity should consider genetic factors and the athlete’s mindset, as genetic and psychological aspects influence athletic success. It is important to remember that discriminating against individuals based on their genetic makeup can significantly affect their participation in sports and overall quality of life. The Universal Declaration on the Genome and Human Rights and the Convention on Human Rights and Biomedicine both emphasize the prohibition of genetic discrimination. However, not all European Union member states have ratified the Convention on Human Rights and Biomedicine [95]. While some countries have signed the convention, indicating their initial approval and intention to consider ratification, they have not completed the ratification process. Signing is a step towards ratification but does not legally bind the country to the convention’s obligations. For instance, Germany, the UK, and Ireland have not ratified the convention. This may require significant legal reforms or raise ethical debates that complicate ratification. The decision of some European Union member states not to ratify the convention has resulted in an inconsistent application of bioethical standards across Europe. As a result, differences emerge in the management of bioethical issues. While non-ratifying states may still safeguard human rights in medicine through their national laws, the ratification of the convention seeks to establish a unified framework for consistent protections. Additionally, ratification can facilitate international cooperation on bioethics and human rights issues, significantly benefiting participating states. Even for countries that have not ratified the convention, its principles may indirectly influence policy and legal developments as a reference point for national legislation. In summary, The Oviedo Convention has not been ratified by all European Union member states, underscoring the varying bioethical standards and human rights protections across Europe. The reasons for non-ratification range from legal and ethical concerns to national sovereignty issues. Nevertheless, the convention continues to be crucial in advancing human rights and ethical norms in biomedicine across Europe.

### 3.6. Ensuring Informed Consent and Data Protection

Genetic research that explores the connection between genotype and phenotype has enabled researchers to analyze polymorphic sites to produce and use a variety of genetic tests [96]. These tests, allowed by the Bioethics Convention for medical diagnostics, enable the detection of genes linked to predisposition for specific disorders and diseases, as outlined in Article 5 of the Universal Declaration on the Genome and Human Rights. It is essential to obtain consent from the bioethics committee at the nearest District Medical Chamber, medical university, or authorized research and development unit when conducting scientific research involving human subjects [97,98]. After receiving approval to conduct the study, adhering to the protocol presented to the bioethics committee is crucial. Primarily, individuals participating in genetic and non-genetic research must provide informed and voluntary consent, as dictated by paragraphs 22–26 of the Declaration of Helsinki (2004) and Article 39 of the Polish Constitution, which states, “No one can be subjected to scientific or medical experimentation without freely given consent”.

It is important to remember that participants have the right to withdraw from the research at any point without facing any consequences [99], and their information will be kept confidential [100,101]. Genetic research results must be protected, following the general rules for safeguarding medical data (Biomedical Convention, Article 10). It is crucial to establish the duration of storage of samples and ensure their proper disposal after the project is finished. The Universal Declaration on the Genome and Human Rights (Article 7) emphasizes strict protection rules for genetic data, as it contains information about a person’s and their relatives’ future. As for sports, WADA and the General Data Protection Regulation (GDPR) of the European Union also protect genetic data, ensuring confidentiality and privacy for individuals undergoing genetic testing.

Predictive genetic tests should only be conducted for medical and scientific reasons. They should be confidential and paired with genetic counseling. Assessing the probability of a condition, especially a multifactorial one, necessitates a detailed report and access to professional assistance [102,103].

The genetic information acquired can often be used to introduce management strategies that minimize the risk of developing certain conditions. For instance, gene therapy [104] can be used without objections in treating injuries and trauma [105,106]. These therapies can accelerate the healing process and reduce the risk of complications after injuries in sports. An early diagnosis of a predisposition and adherence to treatment recommendations can be both curative and preventive [107]. However, challenges arise when a gene leading to an inevitable incidence of a particular disease is identified. There is a dilemma about disclosing the test results to the subject. On the one hand, informing the individual about their genetic predisposition to a specific disease allows them to make informed decisions about their healthcare and potentially take preventative measures. This knowledge can empower them to seek appropriate medical surveillance and make lifestyle changes that mitigate the impact of the disease. However, disclosing such information can be emotionally overwhelming for the individual. They may experience anxiety, fear, and distress upon learning about their genetic predisposition to a severe illness for which there is no known cure or effective treatment. Furthermore, ethical and legal considerations complicate disclosing genetic test results. There is a delicate balance between respecting the autonomy and privacy of the individual and considering the potential benefits to their family members, who may also be at risk due to shared genetic factors. In addition, concerns about the psychological and emotional impact of receiving this information, including potential stigmatization or discrimination, further complicate the decision-making process. The individual’s right to know their genetic status must be weighed against the potential harm the disclosure could cause [108]. Ultimately, the dilemma surrounding the disclosure of test results for a gene leading to an inevitable disease underscores the complexity of genetic counseling and the importance of navigating these sensitive issues with empathy, understanding, and adherence to ethical guidelines [109].

It is essential to recognize that individuals have the right not to be informed of genetic test results. Everyone has the right to choose to remain unaware of their genetic predispositions. Under the Biomedical Convention [110], employers and insurance companies are prohibited from using diagnostic genetic tests. Nonetheless, these tests are permissible for establishing identity and kinship and conducting DNA testing in criminal proceedings [111]. However, the UNESCO Universal Declaration on the Human Genome and Human Rights (1997) underscores this right, stating that individuals can choose not to know their genetic information, a choice protected to safeguard their psychological integrity and personal decision-making autonomy [112].

The discussion on gene doping is pertinent to the broader ethical considerations surrounding genetic interventions in sports. Gene doping is an offshoot of gene therapy but focuses on enhancing athletic performance rather than restoring functions related to damaged or missing genes, as in traditional gene therapy. However, another suggestion is presented as a minority viewpoint. It suggests that if an athlete is aware of the health risks associated with doping but still chooses to use it, considering the long-term harm versus the immediate advantages, then they should be allowed to do so [113,114]. This viewpoint emphasizes an individual’s right to make informed decisions concerning their bodies and well-being, even when the choices involve substantial risks. While this stance may not align with predominant ethical views, it acknowledges and respects diverse perspectives on personal autonomy and informed consent. Furthermore, if gene therapy becomes widespread, athletes could use it to improve their performance. Thus, to deal with this situation, a solution would be to identify these individuals and establish separate divisions for genetically modified athletes to ensure fair competition [68].

#### Genetic Data Sharing (GDS)

Genomics has a strong tradition of sharing data. Genomic information is distributed through different methods, including public, controlled-access, community-specific, and upon-request sharing. Additionally, data are circulated through various platforms, which can be tailored for specific data types or designed as universal repositories to accommodate multiple data types, along with investigator-specific solutions [115]. A question arises about whether the genetic data are easily accessible. A good example is The Human Genome Project. In 2000, the first-ever web-based tool for exploring the human genome was built, sharing the rough draft on the Internet just 11 days after completing the task. However, the broken promise that undermines the Human Genome Project is the failure to share and access genomic data effectively. Despite the initial commitment to make human genome sequences available in public databases with no delays or exceptions, the explosion of data has led to the development of numerous custom-built databases with various rules for access and no standard data formatting. This has created a situation akin to the “Tower of Babel”, making it difficult for researchers to find and use the data. Additionally, there are barriers to uploading and downloading data, and it can be challenging for journal editors and funding agencies to monitor whether scientists adhere to their agreements. This hinders the potential for discoveries and collaborations in the field of genomic research [116].

Another issue relates to direct-to-consumer genetic testing (DTC-GT) services like 23andMe and AncestryDNA, which provide individuals with access to their genomic data and aggregate population-level data. These companies may also share de-identified data with researchers. Ancestry’s website states that it currently holds more than 10 million genetic information records in its databases. In 2018, GlaxoSmithKline’s decision to use data from 23 and Me’s databases to identify and choose pharmaceutical targets sparked controversy over consumer privacy in the media [117].

While genetic data sharing has advanced significantly, the practice has challenges and ethical considerations. The various regulations and diverse data dissemination methods highlight the complexity of responsibly managing genetic information. Although efforts to provide open access to genomic data, such as those initiated by the Human Genome Project, have made substantial progress, a significant gap between the ideal of seamless data sharing and the reality researchers face still needs to be addressed. As we navigate these issues, it is questionable whether the intersection of technology, privacy, and ethics in genomic data sharing is straightforward. How do we reconcile the need for open scientific collaboration with the imperative to protect individual privacy? What frameworks and safeguards are necessary to ensure that genetic data are used responsibly and equitably? These questions remain central to the discourse on genetic data sharing, inviting ongoing reflection and dialogue. The future of genomic research will likely be shaped by how we address these challenges, balancing innovation with ethical considerations.

### 3.7. Patentability and Intellectual Property Issues

There is ongoing debate regarding the patentability of genetic research findings [118,119], including sports [120]. For example, The Genetic Technologies Corp. has obtained a patent for the alpha-actinin-3 (*ACTN3*) gene test in the United States (US 7,615,342 B2; issued 10 November 2009) [121].

The controversial issue revolves around whether granting patents for naturally occurring DNA sequences in the human genome is appropriate [122]. This debate involves various stakeholders, including lawyers, biologists, geneticists, philosophers, ethicists, and business professionals. The legitimacy of granting a patent for a segment of the human genome as an invention is a point of contention. It is debatable whether this kind of information can be categorized as intellectual property rights or, more specifically, as industrial property rights. The debate has been about whether identifying naturally existing biological material should be classified as a discovery. As mentioned, DNA can be documented in patents [123]. These can include the entire gene or a portion of it, such as a promoter; an enhancer, which is a sequence of nucleotides supporting or regulating transcription; an expressed sequence tag (EST); the entire gene in cDNA form; a single mutation causing a specific disease; and a polymorphism (an individual DNA variation not causing disease, e.g., an SNP-type single nucleotide polymorphism) [124]. The development of genetic testing in sports has led to the creation of diagnostic tests that are now used globally. Patents related to the commercial use of tests on specific populations have also emerged (e.g., a method for identifying a genetic predisposition in South Africans to soft tissue injuries involving screening for specific gene polymorphisms in, e.g., *COL51* and *COL12A1* genes) [125].

### 3.8. Ethical Considerations Surrounding the Use of Genetic Technologies in Sports

The use of genetic technologies in sports presents a range of ethical considerations that encompass fairness, health, privacy, and the integrity of competition. In the future, advancements in genotyping may allow for tailored training and strategies based on athletes’ genetic predispositions. Gene doping, understood as the non-therapeutic use of genes or cells to enhance physical performance, is already considered illegal by the WADA. However, these techniques may become more advanced and complex to detect, requiring new regulations and countermeasures. Ethical and legal controversies also surround the potential use of techniques like CRISPR for genetic modifications [126] in sports, including gene doping. New regulations and countermeasures will be necessary as these techniques advance. Ethical discussions and updated rules will be crucial to balance scientific progress with human rights and ethical standards. One of the primary concerns is the potential for genetic enhancement to create an uneven playing field, where athletes with access to genetic modifications may gain unfair advantages. Genetic testing for talent identification raises worries about genetic determinism, potentially overlooking the role of effort and training in athletic potential. Furthermore, the long-term health implications of genetic modifications are mainly unknown, posing severe risks to the athletes’ well-being. Medical professionals must prioritize the health and safety of athletes over performance enhancement when administering genetic technologies.

Consent and autonomy are critical ethical considerations, as athletes must be fully informed and not feel coerced into undergoing genetic modifications or testing. Protecting athletes’ privacy and confidentiality in collecting and storing genetic information is crucial to prevent discrimination or stigmatization.

Ethical concerns also arise around the misuse of genetic technologies, such as gene doping, which undermines the integrity of sports and presents challenges in detection and regulation. Distinguishing between legitimate therapeutic uses of genetic technologies and performance enhancement is ethically complex and requires careful consideration.

The societal impacts of genetic enhancement in sports, including its potential normalization and effects on youth sports, raise concerns about broader societal acceptance and the developmental focus of young athletes.

Enforceable guidelines and policies are essential to govern the use of genetic technologies in sports. They emphasize the need for international consistency to maintain a level playing field and protect athletes globally. The multifaceted ethical considerations surrounding genetic technologies in sports require careful deliberation and robust regulatory frameworks to balance potential benefits with protecting athletes’ rights and the spirit of sports.

Ethical considerations surround the use of genetic technologies in sports. While genetic testing has proven to be valuable in clinical medicine, there are no scientific grounds for using genetic testing for athletic performance improvement, sport selection, or talent identification [22].

### 3.9. Human Genome Research in Sports: Implications of Genetic Advancements in Athletics and the Integrity of Sports

The potential implications of genetic advancements in athletics and the overall integrity of sports closely intertwine with the ethical aspects of human genome research in sports.

Obtaining informed consent from athletes is crucial. This ensures that they completely understand the risks, advantages, and long-term effects of participating in genetic research. Athletes should participate voluntarily without pressure from coaches, sponsors, or sports organizations.

Privacy and confidentiality also play a significant role. Protecting athletes’ genetic information is crucial to prevent misuse and ensure that they are not stigmatized or discriminated against based on their genetic data.

Equity and fairness are essential considerations. Athletes should have fair access to genetic technologies, and measures need to be in place to prevent genetic enhancements from creating disparities in performance and affecting fair competition in sports.

Genetic advancements can affect fairness and equality, potentially giving some athletes unfair advantages and increasing inequality in sports. There are also concerns about the health and safety of athletes, as genetic modifications may have unforeseen consequences and could lead to pressure to undergo enhancements, compromising their well-being and autonomy.

The integrity of sports is at stake, as genetic enhancements might lead to skepticism about the authenticity of athletic achievements and impact public trust. Furthermore, regulatory and legal issues, such as detecting genetic enhancements and establishing international standards, are challenging but essential to prevent genetic doping and protect the integrity of sports.

Genetic information holds enormous potential in athletic training and performance. However, the ethical and practical challenges of harnessing genetic data for these purposes are significant. With the rapid advancements in genetic technologies, new avenues for understanding and optimizing athletic potential and performance have emerged. According to recent research, over 100 companies provide a range of direct-to-consumer genetic tests (DTC-GTs) related to health. These tests cover nutrigenetics, pharmacogenetics, athletic performance, and overall disease risk [127]. DTC-GTs have become increasingly popular and are easily purchased through the Internet, independent of a physician referral or approval for testing, allowing the retrieval of genetic information outside the clinical context [128]. After receiving their genetic test results, individuals might choose to have these results interpreted by an independent third-party service or professional. These third-party interpreters analyze the raw genetic data to provide additional insights, context, or recommendations that the original testing company might not offer. However, the popular trend of using DTC-GTs is coupled with the emergence of third-party interpretation services that offer additional genetic data analyses. These developments hold promise for enhancing athletic training and performance while also raising significant ethical concerns that we must carefully consider within the context of sports genetics.

The commercial aspect of DTC genetic testing means that private companies often handle individuals’ genetic information, potentially prioritizing profits over data protection. Third-party interpretation services further complicate privacy issues by sharing sensitive genetic data with additional entities. The risk of data breaches or unauthorized access to genetic information poses a significant concern. Athletes and consumers must understand how their data are stored, used, and shared and whether it is safeguarded from potential misuse. For example, in a study aiming to explore privacy and risk perceptions, only 4 out of 20 consumers had immediate privacy concerns. Moreover, most participants stated that they did not consider their DNA particularly sensitive personal information [129]. This suggests that they might not consider their genetic data as private or personal as other types of information, such as financial records or personal correspondence. This is in line with another study, where 20 out of 40 participants recognized that there could be privacy issues. The questionnaire respondents did not perceive privacy concerns as overwhelming or prohibitive. This could imply that the individuals believe that the benefits of the situation outweigh the privacy risks or that the risks can be managed or mitigated [130]. Meanwhile, although generally, DTC-GT companies have privacy policies; the terms of use and privacy policies of DTC-GT are sometimes known as “click-wrap” or “browse-wrap” agreements [117]. A click-wrap agreement is an online contract where users show their agreement to the terms and conditions by clicking a button or checkbox. On the other hand, a browse-wrap agreement is an online contract where the terms and conditions are available on the website but do not require explicit action from the user to accept them. Users are considered to accept the terms simply by using the website or service. Providers often use these terms of service agreements, which are legal contracts, instead of informed consent procedures. However, this safeguards consumers from potential physical, psychological, and social harms linked to personal genome testing and supports independent decision-making when considering the testing options [131]. Meanwhile, healthcare professionals voiced concerns that consumers’ information would not be confidential [132]. Whether individuals should obtain informed consent for personal genome testing, even commercially offered, is debatable.

Another aspect is transparency. Some recommendations address scientific aspects, such as conveying the reliability of evidence supporting different genetic forecasts. However, there are worries about the openness of direct-to-consumer genetic testing companies regarding their operations and standards. This lack of transparency has drawn the attention of policymakers and the biomedical industry [133]. On the other hand, DTC testing companies provide information about their testing methods and ensure privacy as the highest priority. Moreover, The Future of Privacy Forum, a think tank and advocacy group based in Washington DC, held discussions with major direct-to-consumer genetic testing companies (23 and Me, Ancestry, Helix, MyHeritage, and Habit) to establish best “voluntary” guidelines for the use and security of genetic information. They determined that the most effective consumer privacy guidelines should (1) ensure transparency regarding the use, collection, and sharing of consumer genetic information; (2) offer consumers choices regarding consent for participation in research and the destruction of their DNA samples; and (3) strengthen consumer protections to guarantee that their genetic information is shared in compliance with relevant laws and with the utmost discretion (such as robust data security measures and stringent legal processes for disclosure) [117]. The recent National Academies workshop discussed the potential merger of DTC-GT and physician-ordered testing as technologies evolve. Some experts highlighted the need for unified risk assessment and standardization in genetic testing practices. In contrast, others raised concerns about the impact of such a merger on healthcare systems and individual decision-making. Alternatively, maintaining DTC genetic testing as a parallel system could offer benefits such as more access to personal health data but may also lead to continued differences in oversight and support [134].

Considering the implications and concerns surrounding direct-to-consumer genetic testing in the context of sports genetics, it is of interest to address some critical questions. Is it appropriate to subject a child or young athlete to direct-to-consumer genetic testing to determine or modify their training regimen or to identify gifted ones? Is it necessary to create a benchmark genetic profile of an outstanding athlete, enabling the development of comprehensive solutions for obtaining early and accurate information regarding individual predispositions? Such projects might allow for acquiring early and accurate information regarding individual predispositions for performing primarily aerobic or anaerobic exertion. Coaches and athletes can utilize these characteristics in professional sports training to select disciplines and types of training to maximize the chances of achieving athletic success for a given athlete. Simultaneously, such projects might prevent sports performance and kill self-esteem [135]. However, creating such profiles involves significant ethical and practical challenges. It requires ensuring that the data are used responsibly, protecting the privacy of athletes, and avoiding potential misuse or discrimination based on genetic information. Moreover, a benchmark profile might inadvertently lead to exclusion or bias, where athletes not fitting the profile are overlooked.

### 3.10. Seeking New Study Areas Not Yet Addressed

Exploring the potential long-term health implications of genetic modifications in athletes could involve studying the effects of specific genetic enhancements on overall health, longevity, and susceptibility to certain medical conditions. Further investigation into the psychological and social impacts of genetic testing and enhancements in sports could provide valuable insights into athletes’ experiences and perceptions and the societal implications of genetic modifications in sports. As gene editing technologies advance, exploring the ethical implications of specific gene editing techniques, such as CRISPR-Cas9 [66], in the context of sports genetics would be beneficial. Research could examine the intersection of genetic diversity and inclusion in sports, exploring how genetic testing and talent identification processes affect equitable athletic opportunities for individuals from diverse genetic backgrounds. Further studies could assess the effectiveness of regulatory frameworks and governance structures in addressing the ethical and legal considerations of genetic technologies in sports, examining international and national policies and their implications for athlete rights and genetic privacy. Investigating public perceptions of genetic advancements in sports and developing educational initiatives to promote informed discussions about genetics, ethics, and sports could be valuable areas for future research. Exploring these new study areas could contribute to a more comprehensive understanding of genetics’ ethical, social, and scientific dimensions in sports and athletic talent identification.

## 4. Conclusions

The rapid advancements in genetics in sports highlight the need for continuous ethical scrutiny. As genetic research in sports progresses, it is crucial to balance the benefits of genetic advancements with ethical considerations. It is important to note that genetic research in sports and exercise is still in its initial phases. However, with the rapid advancements in genetics and its impact on sports, it is vital to consider the ethical implications of potential outcomes at every step. This includes ensuring fair competition, protecting athletes’ rights, and preventing the illicit use of genetic technologies in sports. Genetics has substantially progressed and transformed, particularly in the past few decades.

The potential for unethical behavior, such as using genetic information to influence athletic performance, requires constant updates to legal frameworks to prevent misuse. Therefore, there should be a greater focus on allocating resources to prevent and address the illicit use of molecular biology advancements in sports. Additionally, the legal framework for this type of research should be regularly updated. The focus should be on the responsible application and continuous evaluation of the ethical implications in this evolving field. While genetic advancements offer the opportunity to enhance the diagnosis and treatment of genetic diseases, they also present the risk of unethical behaviors, such as influencing coaches, advisors, and doctors who may rely on genetic information to judge an athlete’s performance or health.

## Data Availability

The original contributions presented in the study are included in the article. Further inquiries can be directed to the corresponding author.
